# Effect of Quercetin on Hepatitis C Virus Life Cycle: From Viral to Host Targets

**DOI:** 10.1038/srep31777

**Published:** 2016-08-22

**Authors:** Ángela Rojas, Jose A. Del Campo, Sophie Clement, Matthieu Lemasson, Marta García-Valdecasas, Antonio Gil-Gómez, Isidora Ranchal, Birke Bartosch, Juan D. Bautista, Arielle R. Rosenberg, Francesco Negro, Manuel Romero-Gómez

**Affiliations:** 1 UCM Digestive Diseases, Virgen Macarena-Virgen del Rocío University Hospitals and CIBERehd, Institute of Biomedicine, University of Sevilla, Sevilla, Spain; 2 Unit for the Clinical Management of Digestive Diseases, Hospital Universitario Valme de Sevilla, Sevilla, Spain; 3 Division of Clinical Pathology, University Hospital, Geneva, Switzerland; 4 University Paris Descartes, EA 4474 “Hepatitis C Virology”, France; 5 Inserm U1052, Cancer Research Centre, University of Lyon, France DevWeCan Laboratories of Excellence Network (Labex), Lyon, France; 6 Biochemistry and Molecular Biology, Faculty of Pharmacy, University of Sevilla, Spain; 7 Division of Gastroenterology and Hepatology, University Hospital, Geneva, Switzerland

## Abstract

Quercetin is a natural flavonoid, which has been shown to have anti hepatitis C virus (HCV) properties. However, the exact mechanisms whereby quercetin impacts the HCV life cycle are not fully understood. We assessed the effect of quercetin on different steps of the HCV life cycle in Huh-7.5 cells and primary human hepatocytes (PHH) infected with HCVcc. In both cell types, quercetin significantly decreased i) the viral genome replication; ii) the production of infectious HCV particles and iii) the specific infectivity of the newly produced viral particles (by 85% and 92%, Huh7.5 and PHH respectively). In addition, when applied directly on HCV particles, quercetin reduced their infectivity by 65%, suggesting that it affects the virion integrity. Interestingly, the HCV-induced up-regulation of diacylglycerol acyltransferase (DGAT) and the typical localization of the HCV core protein to the surface of lipid droplets, known to be mediated by DGAT, were both prevented by quercetin. In conclusion, quercetin appears to have direct and host-mediated antiviral effects against HCV.

The hepatitis C virus (HCV) is an enveloped, positive strand RNA virus belonging to the *Flaviviridae* family^1^ with 7 major genotypes^2^. The disease spectrum ranges from acute to chronic hepatitis, cirrhosis and hepatocellular carcinoma.

The HCV life cycle is tightly linked to the host cell lipid metabolism. Mechanisms linking HCV infection and lipid metabolism include^3^,^4^: (a) HCV circulates as lipid-enriched particles, referred to as lipoviroparticles (LVPs)^5^; (b) LVPs are the HCV particles of highest infectivity due to their association with lipoproteins^6^; (c) several receptors involved in lipid uptake, e.g. low-density lipoprotein (LDL)-receptor, Niemann-Pick C1-like 1 (NPC1L1)^7^ and SR-B1, are implicated in LVP entry into the hepatocyte^8^; (d) HCV assembly occurs in close proximity to lipid droplets (LDs)^9^,^10^; (e) HCV infection promotes accumulation and redistribution of LDs in the perinuclear region^11^; (f) diacylglycerol acyltransferase-1 (DGAT1), an enzyme that synthesizes triglycerides (TG) in the endoplasmic reticulum, interacts with HCV core protein, and is implicated not only in the formation of new LDs but also in the production of infectious HCV^12^; (g) the very low density lipoprotein (VLDL) secretion pathway has been reported to be hijacked by HCV for viral particle secretion^13^.

Treatment with direct acting antivirals (DAA) drugs has dramatically changed outcomes of hepatitis C. Indeed, the sustained viral response (SVR) rates have reached unprecedented levels (>95%)^14^,^15^ without relevant adverse events. However, the price is still one of the major barriers to achieve hepatitis C eradication mainly in low- and middle-income countries^16^.

Several flavonoids such as naringenin and catechin have shown antiviral properties against HCV^17^. Quercetin, a flavonoid present in many components of human diet^18^, has also been reported to have anti-HCV properties by several mechanisms: it has been found to decrease internal ribosomal entry site (IRES) activity^19^, and to inhibit HCV replication^20^ and NS5A-driven IRES-mediated translation of the viral genome^21^,^22^,^23^. Quercetin plays a protective role in diseases such as cancer, coronary heart disease and atherosclerosis because it modulates lipid profile and antioxidant status^24^. Moreover, quercetin modifies eicosanoid biosynthesis, protects LDL from oxidation, prevents platelet aggregation, and promotes relaxation of cardiovascular smooth muscle^25^. Finally, quercetin has been found to inhibit DGAT activity^26^,^27^, an enzyme involved in the assembly step of the HCV life cycle^12^. The main limitation for use of flavonoids in general and quercetin in particular has been low bioavailability requiring orally high doses. Thus, quercetin is widely available, cheap, and has previously demonstrated antiviral activity against HCV. In a phase I dose escalation study, quercetin demonstrated high safety (up to 5 g per day) and antiviral efficacy in hepatitis C patients^28^. The main aims of this study were to further elucidate at which steps of the virus life cycle and by which mechanisms quercetin exerts anti-HCV activity.

## Results

### Effect of quercetin on HCV life cycle in Huh-7.5 cells

**Figure 1:**
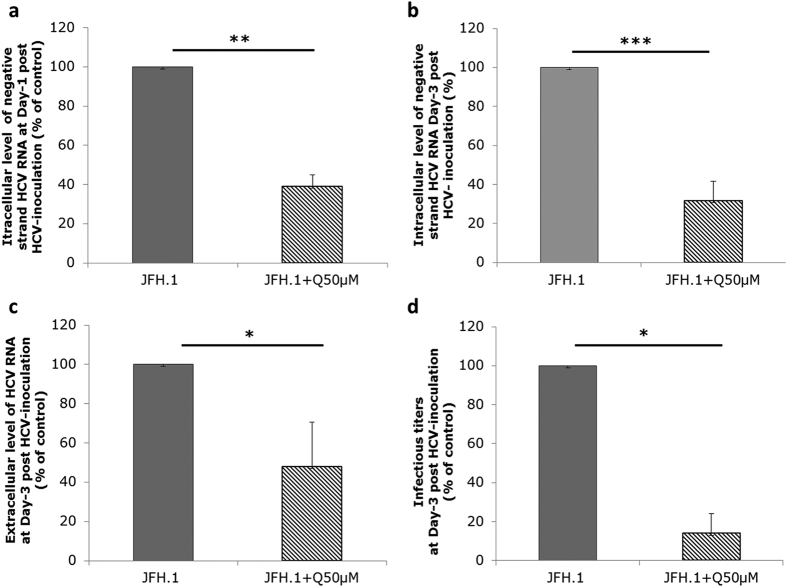
Effect of quercetin on HCV viral life cycle in Huh-7.5.1 cells. Huh-7.5.1 cells were infected with JFH1 for 6 h and then treated with 50 μM of quercetin (JFH1 + Q50 μM), or DMSO as carrier control, for 24 h (**a**) or 72 h (**b,d**). (**a,b**) Cells were lysed for quantification of negative-strand HCV RNA. (**c,d**) Culture supernatant were collected for determination of extracellular HCV RNA level and infectivity titer. Results are expressed as percentage of vehicle control. Data are presented as the mean values ± SD obtained from three independent experiments.

To evaluate the effect of quercetin on HCV genome replication, Huh-7.5 cells were infected with cell culture-produced HCV (HCVcc) of JFH1 strain and treated with 50 μM quercetin, i.e., a concentration at which no toxic effect was observed (Fig. S1A, a and b). Quercetin significantly decreased the intracellular amount of negative-strand HCV RNA, a hallmark of HCV genome replication, assessed at day 1 post-inoculation (61% ± 5.89% inhibition; p = 0.0084) and day 3 post-inoculation (68.38% ± 10% inhibition; p < 0.001) when compared to DMSO-treated infected cells (Fig. 1a,b, respectively). To further examine the impact of quercetin on the HCV life cycle, we evaluated the extracellular production of the viral particles. Cell culture media of Huh-7.5 cells infected with JFH1 were collected 72 h after treatment with 50 μM quercetin for quantification of HCV RNA by a viral load assay, which reflects the production of physical viral particles irrespective of whether they are infectious or not, and determination of infectivity titers by focus-formation assay, which reflects production of infectious viral particles. We observed a decrease of the viral load by 52.08% ± 22.6% (p = 0.016) in the culture medium of cells treated with quercetin compared to DMSO-treated cells (Fig. 1c), which may be a consequence of the quercetin-induced inhibition of HCV genome replication. Most interestingly, however, the infectious titer was even more decreased than the viral load in cells treated with quercetin compared to DMSO-treated cells (86% ± 10% inhibition; p = 0.016) (Fig. 1d). Accordingly, the specific infectivity (calculated as the ratio of infectious titer to viral load) was also decreased by quercetin treatment (around 85% inhibition), suggesting that quercetin not only has an effect on HCV genome replication but also impacts the morphogenesis of infectious particles.

### Effect of quercetin on HCV life cycle in primary human hepatocytes

**Figure 2:**
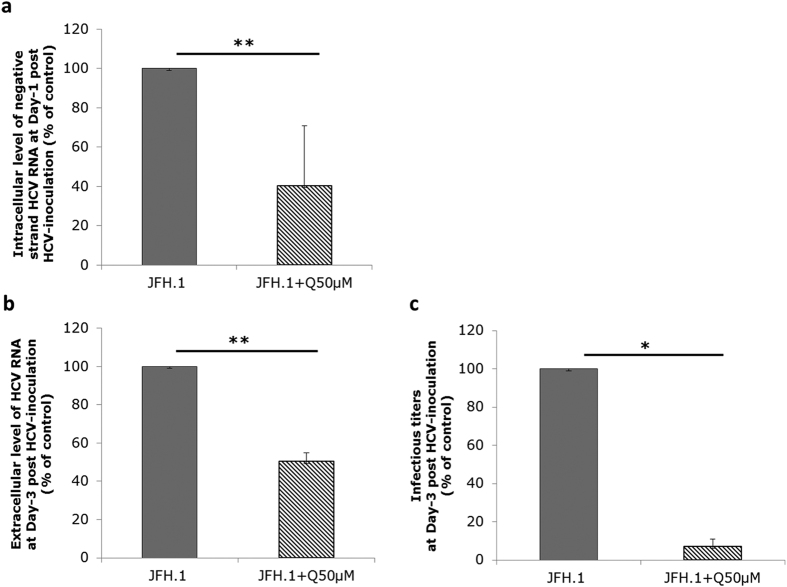
Effect of quercetin on HCV life cycle in PHH. PHH were infected with JFH1 (MOI of 2.5) for 6 h and then treated with 50 μM quercetin (JFH1 + Q50 μM), or DMSO as carrier control, for 24 h (**a**) or 72 h (**b,c**). (**a**) Cells were lysed for quantification of negative-strand HCV RNA. (**b,c**) Culture supernatant were collected for determination of extracellular HCV RNA level and infectivity titer, respectively. Results are expressed as percentage of carrier control. Data are presented as the mean values ± SD obtained from two to three independent experiments.

Although Huh-7 sublines are the most efficient cells for culturing HCV, they display significant differences with normal hepatocytes, especially in the VLDL biogenesis^29^. We thus decided to validate our results using primary human hepatocytes (PHH) as a more physiological cellular model, which allows the production of HCV particles with properties similar to those produced *in vivo*^29^. PHH were inoculated with JFH1 HCVcc and maintained in primary culture in the presence of 50 μM quercetin or DMSO as vehicle control. Intracellular negative-strand HCV RNA assessed at 24 h post-inoculation was significantly decreased in quercetin-treated compared to DMSO-treated PHH (59.20% ± 30.37% inhibition; p = 0.0043) (Fig. 2a). As observed in Huh-7.5 cells, quercetin in PHH not only decreased the viral load but caused an even greater decrease in the infectivity titer (49.69% ± 4.59% inhibition; p = 0.0084 and 92.85% ± 3.69% inhibition; p = 0.039, respectively) (Fig. 2b,c). Hence, the specific infectivity of HCV particles produced in PHH was also significantly decreased by quercetin treatment (91.84% ± 5.35% inhibition; p = 0.037), suggesting that, in PHH as in Huh-7.5 cells, quercetin affects the morphogenesis of infectious particles.

### When applied directly onto HCV particles quercetin reduces their infectivity

**Figure 3:**
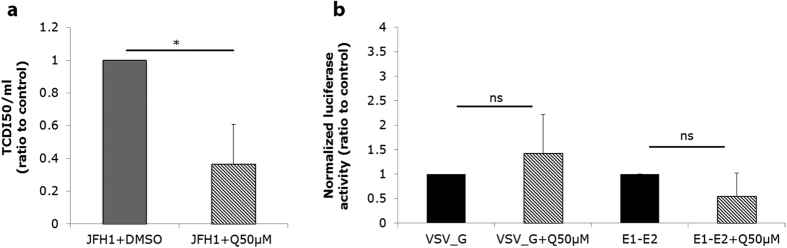
Quercetin reduces the infectivity of HCV particles. (**a**) JFH1-HCVcc virions were incubated in the presence of 50 μM quercetin (JFH1 + Q50 μM), or DMSO as carrier control, for 1 h at 37 °C in cell-free conditions, then used to inoculate Huh-7.5 cells. HCV infectivity was measured 72 h later by the TCID_50_ assay. (**b**) Effect of quercetin on HCV entry step. Huh-7.5 cells were treated with 50 μM quercetin, or DMSO as vehicle control, and transduced with HCVpp, or VSV-Gpp as control, 6 h later. Seventy-two hours post-transduction, a luciferase assay was performed. Results are represented as mean value ± SEM of ratio to control obtained from three independent experiments.

The results obtained so far indicate that quercetin affects at least two distinct steps of HCV life cycle, viral genome replication and production of infectious particles. We next used the HCV pseudo-particle (HCVpp) system to study the effect of quercetin on the HCV entry step. Huh-7.5 cells were treated with quercetin 6 h before transduction with HCVpp and the luciferase activity was measured after 72 h. The results showed that quercetin did not affect HCVpp uptake (Fig. 3b). Nevertheless, we considered the hypothesis that quercetin might exert a direct effect on the virion itself. To test this, JFH1-HCVcc particles were incubated in the presence of 50 μM quercetin, or DMSO as control, for 1 h at 37 °C before being used to inoculate Huh-7.5 cells, and infectivity was assessed by TCID_50_ assay. We observed that quercetin significantly reduced HCV infectivity (63.44% ± 24.44% inhibition; p = 0.018), suggesting that quercetin acts directly on HCV, modifying the integrity of viral particles (Fig. 3a). We conclude that although quercetin does not affect HCV entry when applied onto target cells, it does affect HCV infectivity when applied directly onto the virions.

### DGAT1, a possible candidate as target of quercetin in HCV infection context

**Figure 4:**
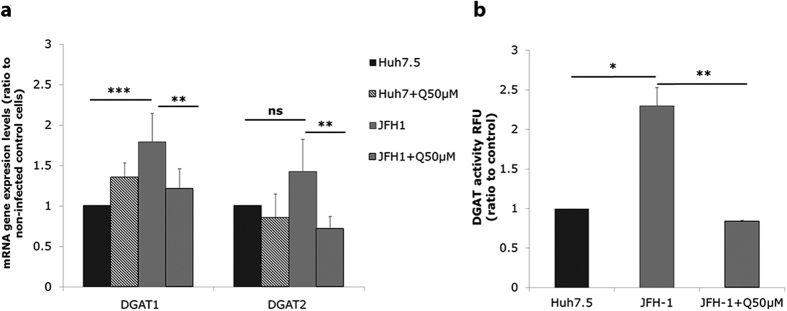
Effect of quercetin on DGAT gene expression level and activity. Huh-7.5 cells were infected with JFH1 (1 MOI) for 72 h in presence or not of 50 μM quercetin. (**a**) DGAT mRNA expression levels were determined by RT-PCR. Results were normalized using GAPDH and DMSO-treated non-infected cells were used as reference. *p < 0.05; **p < 0.01 and ***p < 0.001 (**b**) DGAT activity (ratio to DMSO-treated cells, standardized to non-infected cells). *p < 0.05 and **p < 0.01. Data are the mean value ± SD obtained from three independent experiments.

We investigated the effect of quercetin on the expression of key genes involved in lipid metabolism (Fig. S2, Fig. 4a). Results showed that genes implicated in lipid neosynthesis or uptake [Low-density lipoprotein receptor (LDLr), Fatty acid synthase (FASN), Acetyl-CoA carboxylase (ACC), and Sterol regulatory element-binding transcription factor 1 (SREBP1c)] were upregulated in Huh7.5 infected cells. DGAT1 mRNA expression was also significantly increased upon HCV infection (1.79 fold ± 0.35; p < 0.001) (Fig. 4a). DGAT2 mRNA levels also tended to increase in infected cells, albeit the difference with non-infected cells was not significant (Fig. 4a). On the contrary, Microsomal triglyceride transfer protein (MTP) and Apolipoprotein B (ApoB) tended to be decreased by the virus (Fig. S2). The HCV-induced increase of FASN, LDLr, ACC, SREBP1c (Fig. S2) and DGAT1 (Fig. 4a) mRNA levels could be counteracted by treatment with 50 μM quercetin. As well, MTP gene expression level tended to be lowered by quercetin (Fig. S2).

Taking into account the role of DGAT in the HCV viral life cycle^12^ and our previous data^30^, we decided to further investigate the impact of quercetin on DGAT role on HCV infection context.

DGAT are key microsomal enzymes in TG biosynthesis and DGAT1 is a key host factor for HCV infection. Indeed, DGAT1 interacts with HCV core protein, which forms the viral nucleocapsid, and is required for the trafficking of core protein to LDs, which is essential for infectious virion production^12^. Quercetin was previously reported to reduce TAG synthesis, partly *via* an effect on DGAT activity^26^,^27^,^31^,^32^.

These results were then confirmed at the level of DGAT activity, with a 2.29 ± 0.23-fold increase in HCV-infected Huh-7.5 cells as compared to non-infected control cells (p = 0.015) (Fig. 4b). Interestingly, the HCV-induced increased activity of DGAT could be fully prevented by treatment of infected cells with quercetin (Fig. 4), suggesting that DGAT is one of the targets of quercetin in HCV-infected Huh-7.5 cells.

### Effect of quercetin on LD size and subcellular localization of HCV core protein

**Figure 5:**
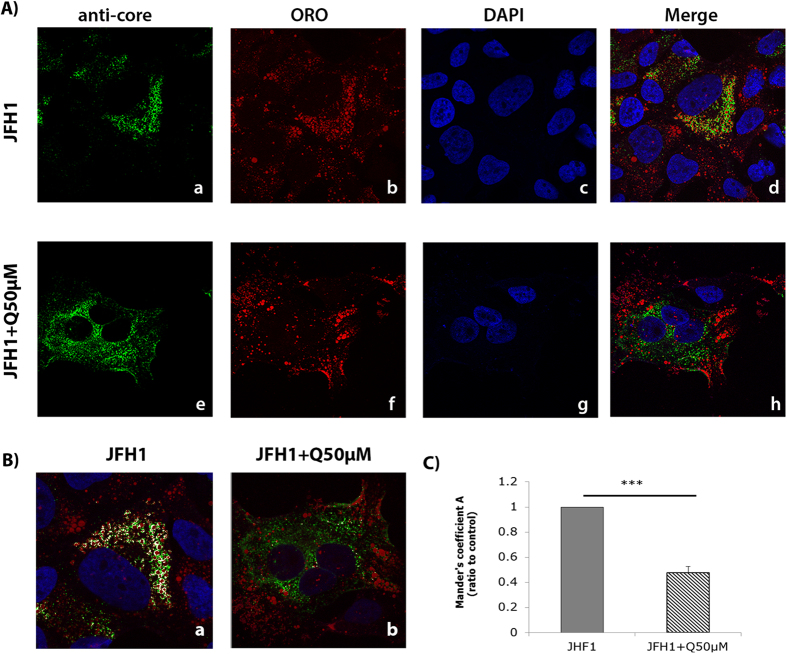
Effect of quercetin on the HCV core protein subcellular localization. (**A**) Huh-7.5 cells were infected with JFH1 and further treated with either the vehicle (DMSO), (a) or with quercetin for 48 h (e). Core protein was detected using a specific antibody (a–e), LDs were stained with ORO (b–f) and nucleus with DAPI (c-g). Overlay images are shown in d and h. Images were taken using a confocal microscope (LSM700 Meta, Zeiss) equipped with a 63x objective. (**B**) Colocalization pictures. Core protein and LDs colocalization were analyzed using the Imaris software 3D Colocalization. The presence of white pixels represents the green pixels colocalized with the red pixels. **(C)** Colocalization statistical analysis obtained by Imaris software was analyzed using the Mander’s coefficient value. The results are means ± SD obtained from three independent experiments (25 cells were analyzed) (***p < 0.001).

Many reports have suggested that HCV assembles at the surface of LDs^9^,^33^. We evaluated the effect of quercetin on LD morphology in Huh-7 cells by Oil Red O (ORO) staining (Fig. S3A, a and b). Morphometric analyses showed that quercetin decreased the mean LD radius by 22.14% ± 8.95% (p = 0.0013). As a consequence, area and volume were also decreased by 39.49% ± 17.72% (p = 0.0019) and 49.60% ± 26.28% (p = 0.003), respectively (Fig. S3B). As DGAT1 has been shown to be essential for the recruitment of the HCV core protein to the LDs^12^, we assessed whether quercetin could have some repercussions in core localization around LDs. To this goal, JFH1-infected Huh-7.5 cells were treated with quercetin for 48 h and the subcellular localization of the HCV core protein was investigated by immunofluorescence. As shown in the Fig. 5, while, as expected, the core protein of JFH1 nicely localized around the LDs in DMSO-treated cells (Fig. 5A-d) (as demonstrated by the presence of white pixels in the co-localization image, Fig. 5B-a), it displayed a more diffuse and punctuated pattern throughout the cytoplasm of quercetin-treated cells (Fig. 5A-h,B-b). Statistical analysis of co-localization confirmed that quercetin significantly disrupted the localization of HCV core protein to the surface of LDs (Fig. 5C).

## Discussion

**Figure 6:**
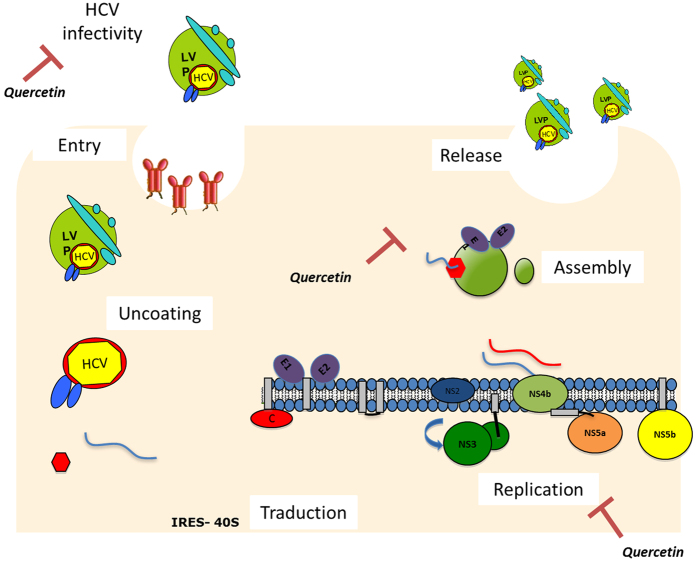
Effect of quercetin on HCV life cycle. Quercetin inhibited viral genome replication and infectious particle morphogenesis. Moreover, it reduced the infection rate when applied directly onto the virions. Moreover, the infectivity capacity of the newly produced viral particles was reduced by quercetin treatment.

In this study, we showed that quercetin modifies HCV life cycle at several steps (Fig. 6): it i) inhibits HCV genome replication; ii) affects the morphogenesis of infectious particles, thus decreasing HCV specific infectivity; iii) affects the virion integrity when applied directly onto HCV particles; and iv) hampers the localization of HCV core protein to LDs. In addition to the virus itself, we identified DGAT1, a key host factor for HCV infection, as one of the target of quercetin. Quercetin is a ubiquitous flavonoid that has been reported to display antiviral activity against several viruses. In particular, it was shown to reduce the replication of several respiratory viruses^34^,^35^,^36^. In the case of HCV, quercetin decreases HCV particle production by partly blocking the ability of NS5A to facilitate viral cap-independent translation^19^. In addition, Bachmetov *et al*. recently identified quercetin as an active substance responsible for the inhibition of NS3 protease activity, thus decreasing HCV production^20^. In our study, the effect of quercetin on HCV genome replication and infectious virus morphogenesis in hepatoma cells was corroborated in PHH, which support production of viral particles whose properties are similar to those found in serum of patients with hepatitis C^29^. Our observation that the specific infectivity is decreased by quercetin suggests that the drug specifically impairs the morphogenesis of the most infectious particles, i.e., LVPs.

Quercetin modulates the activity of key enzymes in lipid metabolism such as DGAT1, ACC and MTP^26^,^27^. In addition, quercetin intake prevents the lipid accumulation in the liver of mice fed with a high fat diet^37^. Our data indicate that in the context of HCV infection, quercetin prevents HCV-induced modulation of mRNA levels of several genes involved in the lipid biogenesis, secretion and uptake. In addition, our results indicate that quercetin avoids the increase of DGAT protein activity induced by HCV, suggesting that DGAT could be a target of quercetin, albeit not exclusive, in lipid metabolism. It is well known that DGAT1 and DGAT2 catalyze the final step of triglyceride biosynthesis and are essential in LD biogenesis^38^. In our study, quercetin reduced LD size. LDs have been proposed to serve as platform for HCV assembly, thus quercetin-induced reduction of the activity of DGAT could in turn decrease the LD size by reducing the neutral lipid content and consequently the LD membrane area available for HCV assembly. Another possible explanation for the effect of quercetin on HCV morphogenesis comes from our observation that quercetin disrupted the localization of the core protein around LDs, probably via decreased DGAT activity^12^. A phase I clinical trial reported safety and antiviral effect of quercetin in patients with chronic hepatitis C^28^. High doses of quercetin were well tolerated and the authors suggested that it could be used to prevent relapse. In our study, we observed that when directly applied onto HCV particles, quercetin modifies their infectivity, suggesting that this drug affects the virion integrity and virulence and may be considered as a coadjuvant in prophylaxy after accidental exposure to HCV to slow down viral infection.

In conclusion, we showed that quercetin targets both viral and host factors, and hence interferes with HCV infectious cycle at different steps. Our results further confirm that HCV hijacks the host lipid metabolism to fulfill its life cycle from assembly to replication steps. Quercetin pre-empts the subcellular localization of core protein to LDs, pointing to a major effect of quercetin on lipid metabolism. Moreover, quercetin is able to decrease HCV infectivity by at least two distinct mechanisms: (i) when applied onto producing cells it affects the morphogenesis of infectious particles, and (ii) when applied onto virions it affects their integrity.

## Materials and Methods

### Reagents, antibodies, plasmids and primers

All reagents, plasmids and primers used in this study are described in the Supporting Information (S1 Table).

### Cell culture

Human embryonic kidney (HEK) 293T and human hepatoma (Huh-7 and Huh-7.5) cells were cultured in low glucose Dulbecco’s modified Eagle’s medium (DMEM) supplemented with 10% fetal bovine serum (FBS), 100 U/ml penicillin, 100 U/ml streptomycin, and 2 mM L-glutamine in a humidified atmosphere at 37 °C and 5% CO_2_. (all from Invitrogen Life Technologies). Experiments with PHH were carried out in accordance with French laws and guidelines. PHH purchased from Biopredic International (Rennes, France) were isolated from ostensibly normal liver tissue obtained from adult patients undergoing partial hepatectomy for the treatment of metastases, and seronegative for HCV, hepatitis B virus and human immunodeficiency virus, with written informed consent by all patients and agreement from the Ministère de l’Enseignement Supérieur et de la Recherche (ID: AC-2013-1754). Experimental protocols were approved by the national French ethics committee (Approval N° D34-172-16). Freshly isolated PHH were seeded at a density of 1.6 × 10^5^ viable cells/cm^2^ onto collagen-coated plates and maintained in primary culture as described previously^29^.

### Quercetin cytotoxicity assay

To test the cytotoxic effect of quercetin, Huh-7.5 cells and PHH were seeded onto 6-well plates and treated with 50 μM quercetin for 72 h. Dimethyl sulfoxide (DMSO) was used as control. Huh-7.5 cells viability was evaluated using the trypan blue exclusion test. To assess cytotoxicity of quercetin on PHH, the activity of lactate dehydrogenase released into culture supernatants was measured with the CytoTox 96R Non-Radioactive Cytotoxicity Assay (Promega), and the ratio of lactate dehydrogenase leakage relative to a carrier control was calculated as previously has been described^39^.

### HCV production, titration and infection assay

To produce HCVcc particles, Huh-7.5 cells *(4* × *10*^*6*^) were electroporated with 5 μg of *in vitro*-transcribed full-length JFH1 RNA^40^ (genotype-2a) using Amaxa cell line nucleofector kit T (260 V, 950 μF Lonza). Culture supernatant was harvested after 72 h, filtered through 0.45 μm pore-sized polyvinylidene difluoride membranes, and titrated by infecting naive Huh-7.5 cells by serial dilutions. Cells were fixed after 72 h with −20 °C methanol and immunostained using an anti-HCV core (C7-50) antibody. Tissue culture 50% infectious dose *(TCID*_*50*_) was calculated as reported^41^. High-titer viral stocks were used to inoculate Huh-7.5 cells or PHH at a MOI of 1 and 2.5, respectively.

### Virological analyses

#### HCV RNA levels- Replication assays

*In vitro* assays were conducted to assess the ability of quercetin to inhibit HCV replication. Huh-7.5 cells and PHH were inoculated with JFH1-HCV. After a 6-h incubation at 37 °C, the inoculum was removed. Cells were washed three times with phosphate-buffered saline (PBS) and replaced in culture medium containing either 50 μM of quercetin or 0.05% DMSO as vehicle control. Replication of HCV genome was assessed by measuring the intracellular levels of negative-strand HCV RNA using a strand-specific quantitative RT-PCR technique described previously^42^. HCV RNA amounts in filtered cell culture supernatants were quantified at 72 h post-infection with commercial standardized viral load assays: the Roche COBAS^®^ TaqMan^®^ HCV Test v2.0 for Huh-7.5 cells or the Abbott RealTime^®^ HCV test for PHH. Infectivity titers were assessed by focus-formation assay and expressed as focus-forming units (ffu)/ml, as previously described^29^,^43^. The effect of quercetin on the specific infectivity of HCV particles produced was calculated as the ratio of infectivity titer to viral load.

### RNA isolation, reverse transcription and quantitative real-time polymerase chain reaction (RT-PCR)

Total RNA was extracted using Trizol^44^. RNA samples were treated with DNaseI. Total RNA was subjected to reverse transcription using commercially available kits (QuantiTect Rev. Transcription Kit; Qiagen, Hilden, Germany) according to the manufacturer’s instructions. Specific primers used are listed in the supplementary table and glyceraldehyde 3-phosphate dehydrogenase (*GAPDH*) was used as reference for normalization. Gene expression levels were determined by Delta Ct method. Fold change was calculated as sample/control ratio in three independent experiments.

### HCV pseudoparticle production

The plasmid phCMV 1b9.9 containing luciferase as a reporter gene was used to produce HCV pseudoparticles (HCVpp) based on the method described^45^. VSV-Gpp entry was evaluated as control. Control pseudoparticles were generated with the VSV-G glycoprotein^46^. Forty-eight hours post-transduction luciferase assay was performed using the Dual-Luciferase assay system kit (Promega) according to the manufacturer’s protocol.

### DGAT activity assay

DGAT activity was measured using *in vitro* assays following the protocol previously described^47^. 50,000 Huh-7.5 cells were seeded in 6-wells plates, infected with JFH1 1MOI and treated with 50 μM quercetin for 72 h. Cells were washed twice with ice-cold PBS, scraped from the tissue culture dish and placed in a 1.5 ml tube to be pelleted by centrifugation at 1000 x g for 2 min. The pellet was re-suspended in 500 μl of 50 mM Tris-Cl (pH 7.6)/250 mM sucrose. Cells were disrupted by extrusion (15 times) through a 27-gauge needle. Cell debris and nuclei were pelleted by centrifugation at 600 x g for 5 min. Total cellular membranes were obtained by centrifuging the supernatant at 100,000 x g for 30 min at 4 °C. The supernatant was removed and the membrane pellet was re-suspended in 50 mM Tris-Cl (pH 7.6)/250 mM sucrose and used for DGAT assays. DGAT activity was measured using the method described by McFie *et al*. based on the using the fluorescent fatty acyl-CoA substrate-{N-[(7-nitro-2-1,3-benzoxadiazol-4-yl)-methyl]amino}(NBD)-palmitoyl CoA and 1,2 dioleoyl-sn-glycerol (DOG) as substrates. The newly synthesized TGs (fluorescent product, NBD-triglyceride) were quantified using a molecular imager (Synergy HT, BioTeK).

### Immunofluorescence and Oil Red O staining

Huh-7.5 cells were seeded onto 24 well plates with coverslips for 24 h and then infected with JFH1. After 6 h cells were treated with 50 μM quercetin for 72 h. Cells were washed twice with PBS and fixed with paraformaldehyde (4%) for 10 min and permeabilized with 0.2% Triton X-100 for 2 min. Cells were incubated with the anti-core antibody (1:300) and Alexa 488-conjugated secondary antibody (1:500). Nuclei were stained with 40,6-diamidino- 2-phenylindole (DAPI) (1:1000) for 30 min at room temperature, and neutral lipids were stained with oil red O (ORO) as previously described^48^. Images were acquired with a confocal microscope (LSM700Meta, Zeiss) using a 63x objective and the surface area of LDs was calculated using the Metamorph software (Molecular Devices Corporation, Sunnyvale, CA).

### Colocalization assessment

Subcellular localization of core around the LDs was analyzed using the Imaris Software. Results were expressed according the Manders Coefficient described as a statistical value that is based on the Pearson’s coefficient with average intensities being taken out of the mathematical expression. This coefficient varies from 0 to 1 with 0 corresponding to non-overlapping images and 1 corresponding to 100% co-localization.

### Statistical analyses

Continuous variables are described as means ± SD or SEM of minimum three independent experiments. The Student *t*-test was used for comparisons between groups. P values P < 0.05 (*) p < 0.01 (**) and p < 0.001 (***) were considered statistically significant.

## Additional Information

**How to cite this article**: Rojas, Á. *et al*. Effect of Quercetin on Hepatitis C Virus Life Cycle: From Viral to Host Targets. *Sci. Rep.* **6**, 31777; doi: 10.1038/srep31777 (2016).

## Supplementary Material

Supplementary Information
