# Supplementary material for: Effect of Quercetin on Hepatitis C Virus Life Cycle: From Viral to Host Targets
Source: Sci Rep. 2016 Aug 22;6:31777. doi: 10.1038/srep31777 (PMC4992894; doi:10.1038/srep31777)
Supplement: Supplementary Information [file srep31777-s1.doc]

**SUPPLEMENTARY INFORMATION**

**Manuscript category:** Research article

**Effect of Quercetin on Hepatitis C Virus Life Cycle: From Viral to Host Targets**

Ángela Rojas^1,2^, Jose A. Del Campo^2^, Sophie Clement^3^, Matthieu Lemasson^4^, Marta García-Valdecasas^1,2^, Antonio Gil-Gómez^1,2^, Isidora Ranchal^2^, Birke Bartosch^5^, Juan D. Bautista^6^, Arielle R. Rosenberg^4^, Francesco Negro^3,7^, Manuel Romero-Gómez^1*^

^1^ UCM Digestive Diseases. Virgen Macarena-Virgen del Rocío University Hospital. Institute of Biomedicine, University of Sevilla, Spain

^2^ UCM Digestive Diseases and CIBERehd, Valme University Hospital, University of Sevilla, Spain

^3^ Division of Clinical Pathology, University Hospital, Geneva, Switzerland

^4^ University Paris Descartes, EA 4474 “Hepatitis C Virology”, France

^5^ Inserm U1052, Cancer Research Centre, University of Lyon, France DevWeCan Laboratories of Excellence Network (Labex), Lyon, France

^6^ Biochemistry and Molecular Biology, Faculty of Pharmacy, University of Sevilla, Spain

^7^ Division of Gastroenterology and Hepatology, University Hospital, Geneva, Switzerland

**Supporting Information**

**Supplementary Table:** List of reagents

**Real-time PCR primers:**

| **Name** | **Cat. no** | **Company/Reference** |
| --- | --- | --- |
| GAPDH | QT00079247 | QIAGEN |
| DGAT1 | QT00087192 | QIAGEN |
| DGAT2 | QT00053445 | QIAGEN |
| LDLr | QT00045864 | QIAGEN |
| FASN | QT00014588 | QIAGEN |
| MTP | QT00043050 | QIAGEN |
| APOB | QT00020139 | QIAGEN |
| ACC | QT00033761 | QIAGEN |
| SREBP1c | QT00036897 | QIAGEN |
| PPAR-gamma | QT00029841 | QIAGEN |

**Primary antibodies:**

| **Protein targeted** | **Host** | **Clone** | **Provider** | **Catalogue number** |
| --- | --- | --- | --- | --- |
| HCV Core | Mouse | C7-50 | Enzo LIfe Science | ALX-804-277 |

**Secondary antibodies**

| **Protein targeted** | **Host** | **Provider** | **Catalogue number** |
| --- | --- | --- | --- |
| HRP-conjugated antimouse | Goat | Biorad | 170-6516 |
| anti-Mouse IgG (H+L) Alexa Fluor® 488 | Goat | ThermoFisher | A11029 |

**Plasmids and HCV constructs**

| **Plasmid** | **Source** | **References** |
| --- | --- | --- |
| pJFH1 (JFH1) | T. Wakita | 1 |

**Other reagents:**

| **Name** | **Provider** | **Catalogue Number** |
| --- | --- | --- |
| Quercetin | HWI ANALALITIK GmbH | HWI 00164 |
| Amaxxa^TM^ Cell Line Nucelofactor^TM^ Kit T | Lonza | VCA-1002 |
| Dimethyl sulfoxide | AppliChem | A1584,0100 |
| COBAS® TaqMan® HCV Test | Roche | 03568555 190 |
| Abbott® Real Time HCV | Abbott Molecular | 01N30 |
| TRIzol® reagent | ThermoFisher | 15596026 |
| QuantiTect Reverse Transcription Kit | Qiagen (Germany) | 205311 |
| NBD-palmitoyl CoA | Avanti Polar Lipids | 810705P |
| 1,2 dioleoyl-sn-glycerol | Sigma (MO, USA) | D0138 |
| Paraformaldehyde (4%) | Affimetrix | 19943 1 LT |
| Triton X100 | Merck | 1.08603 |
| DAPI Nucleic Acid Stain | Molecular Probes - Invitrogen | D1306 |
| Oil-Red-O | Sigma (MO, USA) | O1008-5G |

**Supplementary figures:**

Figure 1


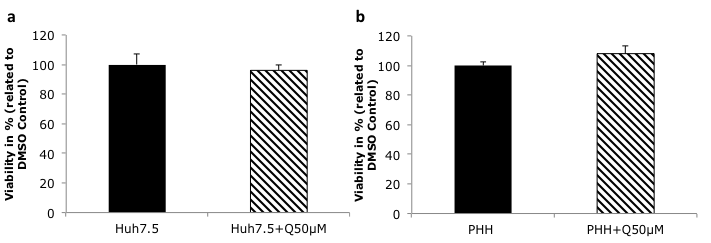


**Figure 1 SUP**. Quercetin effect on cells viability. Huh7.5 **(a)** and PHH **(b)** cells were treated with either DMSO or with 50 μM quercetin for 72h. Huh-7.5 and PHH cells viability was evaluated using the trypan blue exclusion test and by measuring of lactate dehydrogenase activity, respectively. Data are presented as the % mean value of viable cells ± SD obtained from two to three independent experiments.

Figure 2


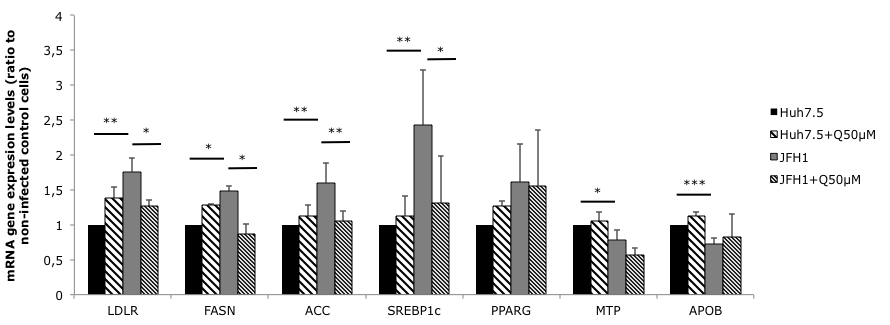


**Figure 2 SUP.** Effect of quercetin on lipid gene expression levels. Huh-7.5 cells were infected with JFH1 (1 MOI) for 72 h in presence or not of 50 μM quercetin. mRNA expression levels were determined by RT-PCR. Results were normalized using GAPDH and DMSO-treated non-infected cells were used as reference. * p<0.05;** p<0.01 and *** p<0.001. Data represent the mean value ± SD obtained from three independent experiments.

Figure 3


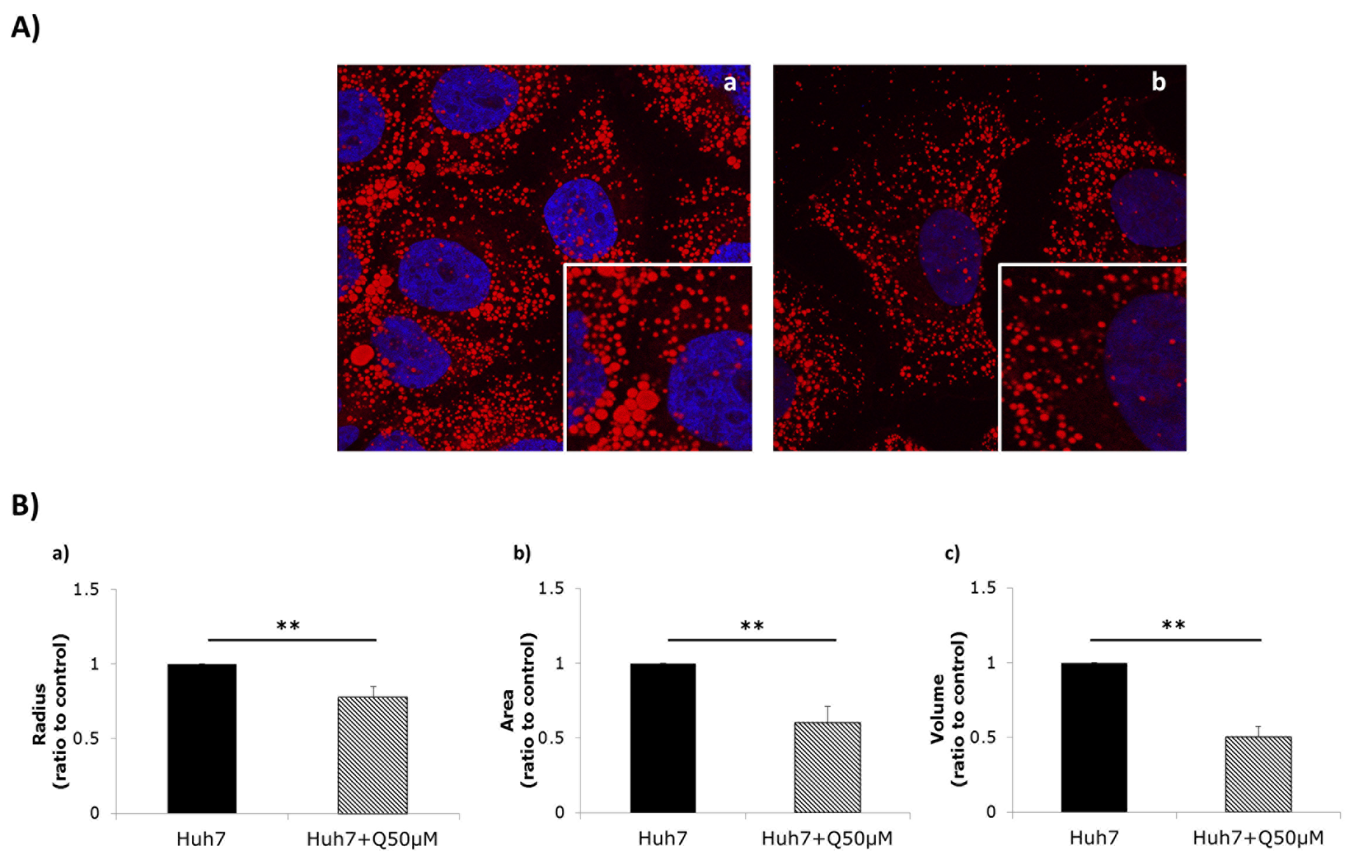


**Figure 3 SUP**. Effect of quercetin on lipid droplet morphology. **A)** Huh-7 cells were treated with either DMSO **(a)** or with 50 μM quercetin **(b)**. LDs were stained with ORO (red) and nucleus with DAPI (blue). Images were captured with a 63x objective using a confocal microscope (LSM700, Zeiss). **B)** LD area, radius and volume were evaluated using the Metamorph software. Fold change was determined using DMSO-Huh-7 cells as control (** p<0.01). Data are the mean value ± SD obtained from three independent experiments.

**References**

1. Kato, T. *et al.*  Efficient replication of the genotype 2a hepatitis C virus subgenomic replicon. *Gastroenterology*. **125(6),** 1808-1817 (2003).
